# Systematic review and meta-analysis for laparoscopic versus open colon surgery with or without an ERAS programme

**DOI:** 10.1007/s00464-015-4148-3

**Published:** 2015-03-24

**Authors:** W. R. Spanjersberg, J. D. P. van Sambeeck, A. Bremers, C. Rosman, C. J. H. M. van Laarhoven

**Affiliations:** 1 Department of Surgery, Radboud University Medical Centre, Huispost 690, Postbus 9101, 6500 HB Nijmegen, The Netherlands; 2 Department of Surgery, Canisius Wilhelmina Hospital, Nijmegen, The Netherlands

**Keywords:** Systematic review, Laparoscopic surgery, ERAS, Meta-analysis, Colon surgery

## Abstract

**Background:**

In recent years, conventional colorectal resection and its aftercare have increasingly become replaced by laparoscopic surgery and enhanced recovery after surgery (ERAS) pathways, respectively.

**Objective:**

To ascertain whether combining laparoscopy and ERAS have additional value within colorectal surgery.

**Methods:**

A systematic review with meta-analysis was performed with two primary research questions; does laparoscopy offer an advantage when all patients receive ERAS perioperative care and does ERAS offer advantages in a laparoscopically operated patient population. All randomised and controlled clinical trials were identified using MEDLINE, EMBASE and Cochrane databases.

**Results:**

Primary search resulted in 319 hits. After inclusion criteria were applied, three RCTs and six CCTs were included in the meta-analysis. For laparoscopically operated patients with/without ERAS, no differences in morbidity were found and postoperative hospital stay favoured ERAS (MD −2.34 [−3.77, −0.91], *Z* = 3.20, *p* = 0.001). When comparing laparoscopy and open surgery within ERAS, major morbidity was significantly reduced in the laparoscopic group (OR 0.42 [0.26, 0.66], *Z* = 3.73, *p* = 0.006). Other outcome parameters showed no differences. Quality of included studies was considered moderate to poor overall with small sample sizes.

**Conclusion:**

When laparoscopy and ERAS are combined, major morbidity and hospital stay are reduced. The reduction in morbidity seems to be due to laparoscopy rather than ERAS, so laparoscopy by itself offers independent advantages beyond ERAS care. Quality of included studies was moderate to poor, so conclusions should be regarded with some reservations.

Bowel resections are one of the most commonly performed surgical procedures today. Colorectal carcinoma is the second most common cancer worldwide, and other benign conditions also often require intestinal surgery.

In recent years, the common procedure and conventional aftercare have become more and more replaced by two new interventions in the care for bowel surgery. First described in 1991, laparoscopic (assisted) surgery has been introduced [1]. Scientific evidence supporting laparoscopic surgery has been accumulating ever since. Large randomised trials and subsequent meta-analyses showed a reduction in hospital stay and reduced morbidity after laparoscopic colorectal oncological resections [2, 3]. Furthermore, oncological radicality was not compromised using the laparoscopic approach [4]. However, the quality of trials included could have biased results [3].

The second treatment modality that has gained popularity in recent years is the “enhanced recovery after surgery” pathway (ERAS). This multimodal treatment modality was first introduced by Kehlet et al. [5] in the mid 1990s and was later developed further by the ERAS working group [6]. ERAS is based on facilitating early recovery after major surgery by diminishing the surgical trauma and the inherent body’s response, thereby preserving bodily composition and organ function. It incorporates 17 different items, ranging from pre-operative counselling and feeding, peri-operative measures to postoperative mobilisation and early feeding. Most of the items used are chosen on the basis of high-grade evidence of clinical efficacy [6]. A recent Cochrane review concludes that ERAS in colorectal surgery is safe and reduces hospital stay, but quality of literature is moderate and major complications are not reduced [7].

Both methods are widely used today, especially in colorectal surgery. However, an additional effect of laparoscopy within ERAS pathways or vice versa has not been investigated as well. To date, no meta-analysis has been published on this subject.

The research question this paper attempts to investigate is whether laparoscopy and ERAS have additional, i.e., synergistic effects in colorectal surgery. To answer this research question, a systematic search of available evidence regarding laparoscopy within ERAS was obtained. This resulted in two separate study groups: first studies looking at laparoscopically operated patients that receive either ERAS or conventional aftercare; second, studies looking at laparoscopic versus open operative techniques while all patients are treated using ERAS. The systematic review and subsequent meta-analysis were performed according to Cochrane guidelines [8] and the manuscript by Mahid et al. [9].

## Materials and methods

The following two research questions were devised before attending to a systematic review;

1. Does ERAS offer an advantage in colorectal surgery when all patients are operated on laparoscopically?
2. Does laparoscopy offer an advantage in colorectal surgery when all patients receive ERAS treatment protocols?

Relevant studies were identified by performing a systematic search of MEDLINE, PubMed, EMBASE and Cochrane databases. No date or language restrictions were applied. A search string was devised for MEDLINE databases;

(((((((“Colon”[Mesh])) OR (“Colonic Diseases”[Mesh]))) OR (((“Intestines”[Mesh])) OR (“Intestinal Diseases”[Mesh]))) OR ((((intestine OR intestines OR intestinal)) OR (bowel OR bowels)) OR (colon OR colonic OR colorectal)))) AND (((((((laparoscopic surgery)) OR (laparoscopy))) OR (“Laparoscopy”[Mesh]))) AND (((enhanced[tiab] AND recovery[tiab] AND surgery[tiab])) OR (“fast track”))).

And this string was changed appropriately for other databases. No restrictions on date or language of publication were applied. The data search was updated in March 2014.

### Inclusion of studies

All prospective trials that included colorectal surgical patients and either compared laparoscopic patients that did or did not receive ERAS or compared patients receiving all ERAS care with or without laparoscopic intervention were eligible for inclusion.

**Fig. 1:**
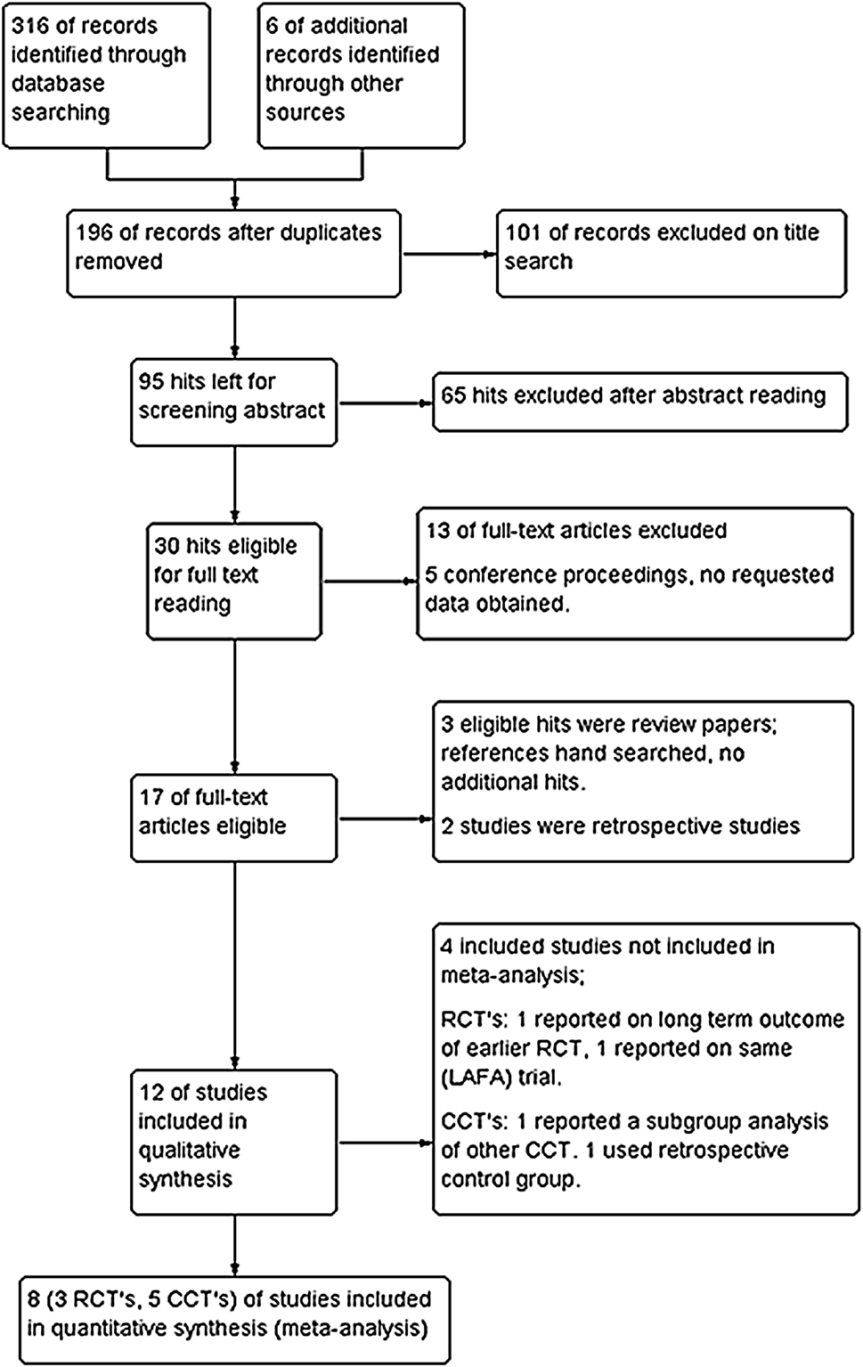
Search strategy

The search was independently performed by two authors (WRS/JvS). Disputes were resolved by discussion, and if necessary, the judgment of the third reviewer (CvL) was considered final. Included studies were also hand-searched for additional relevant references, as were reference lists from identified review papers. The search process is depicted in Fig. 1. Authors from identified conference proceedings were contacted for full data. Also, studies in progress were identified using the Cochrane CENTRAL database. One was identified, and authors were contacted for data. No data were provided.

### Exclusion criteria

Trials had to be published in a peer-reviewed indexed journal. Dual publications or late follow-up from earlier trials was excluded from meta-analysis. Only randomised or controlled clinical trials were eligible for meta-analysis. According to the ERAS working groups’ recommendations, an ERAS programme should incorporate 17 items [6]. The difference in the number of intervention used between the conventional and ERAS groups has to be large enough in order to judge the effect of the intervention named ERAS. In line with the recent Cochrane review [7], we therefore regarded ERAS protocols implementing seven or more ERAS items and conventional protocols implementing no more than two items to be adequate for comparison.

### Other studies

The systematic search for literature also resulted in several studies that could not be included in the meta-analysis [10–17]. Although these studies have an inherent increased risk of bias and did not fulfil inclusion criteria, these studies were read and outcome measures were reported on descriptively.

### Quality assessment and data extraction

All included studies were assessed for quality by using GRADE methodology [18] and analysed by using the GRADE profiler tool, as provided by the Cochrane collaboration. (GRADE profiler, v3.2.2, © Grade working group, 2004–2007). Generation of the allocation sequence, allocation concealment, blinding and follow-up were analysed.

Data extraction was independently performed by using specially designed data extraction sheets. Primary endpoints analysed were complications, both major and minor, readmissions and length of hospital stay. Secondary endpoints included quality of life data, oncological outcome, pain and pain medication and gastrointestinal function.

For each study, patient characteristics, study characteristics, data needed for the methodological quality assessment of the study and the primary and secondary outcomes were extracted according to availability. Data regarding patient characteristics included number of patients in each group, age, gender, BMI and diagnoses of included patients. Data regarding study characteristics included study design, sample size information, inclusion and exclusion criteria of the study, follow-up period, loss to follow-up, surgical experience and information regarding surgical techniques. For each study, data regarding the perioperative interventions in both the ERAS group and conventional group were also extracted.

### Data analysis

In order to attempt a meta-analysis, first clinical heterogeneity was explored. Meta-analysis was performed using Review Manager as provided by the Cochrane Collaboration. [Review Manager (RevMan) (Computer program). Version 5.1. Copenhagen: The Nordic Cochrane Centre, The Cochrane Collaboration, 2011]. Heterogeneity was calculated using Higgins Chi-square test and quantified by measuring *I* ^2^ [19]. A Chi-square test with a *p* value of <0.10 was considered to indicate the presence of heterogeneity, while an *I* ^2^ > 50 % was considered to suggest a marked inconsistency in effect between studies. In case of no discrepancy [and no heterogeneity (*I* ^2^ < 25 %)], the fixed-effect models is presented. In all other cases, the random-effects model was used. For dichotomous data (read-mission rate, morbidity and mortality), OR with 95 % CI was calculated.

Length of hospital stay is divided into primary hospital stay (PHS) and total length of stay (TLOS = PHS + days spent after readmission). Readmissions were defined as readmissions within 30 days of surgery. All included studies reported hospital stay as median with interquartile ranges. Means and SDs were calculated and imputed as described by Hozo et al. [20]. The lower and upper ends of the range were calculated by multiplying the difference between the median and upper and lower ends of the interquartile range by 2 and adding or subtracting the product from the median.

Complications were divided into major and minor complications. Mortality was counted as major complication, but was also assessed separately. Major complications included abdominal sepsis, anastomotic leakage, need for reoperation, persistent ileus, intra-abdominal abscesses, bleeding, burst abdomen (Platzbauch), late incisional hernia and adhesions. Minor complications include pneumonia, wound infection, deep vein thrombosis and urinary tract infection.

## Results

### Search results

The initial search resulted in 316 hits. After removal of duplicates, 196 remained. After screening hits on title, 95 hits remained. Abstracts were obtained, which resulted in 30 remaining hits. Five of these were conference proceedings, and the authors were contacted in order to obtain full data; none complied. The remaining papers were obtained, including three review papers. These were hand-searched and resulted in one additional hit. Full-text analysis resulted in 14 included papers.

Five of the included studies reported on a randomised trial [21–25]; however, two studies reported on the LAFA trial [24, 25], and one study reported on the long-term outcome of an earlier RCT [23].

Six studies were classified as a controlled clinical trial (CCT) [26–31]. Polle et al. [32] performed a case–control study with a retrospective control group and could therefore not be included in primary analysis. Scharfenberg et al. [31] performed a subgroup analysis from data used in Raue et al. [30] and could therefore not be included in analysis. Lloyd et al. reported on a prospective trial including both open and laparoscopic resections, with or without an ERAS protocol. However, for inclusion in this review, only patients within the laparoscopic with or without ERAS were included, because outcome differences in the open group were not reported [28]. The remaining studies were retrospective studies.

### Trials included for primary analysis

From the five included RCTs, three could be included for primary analysis [21, 22, 25]. Vlug et al. used a factorial designed RCT, reporting on four patient groups; open or laparoscopic with or without ERAS care. King [22] reported on an RCT comparing laparoscopic and open surgery within an ERAS programme, as did Basse et al. [21]. The RCTs entered 520 patients in total. 98 patients received traditional care (TC) in open surgery. 142 patients received ERAS care and open surgery, while 109 patients received TC in laparoscopic surgery. Finally, 171 patients received ERAS care and laparoscopic surgery.

In the CCTs included for analysis, 422 patients were entered. No patients received TC in open surgery. 105 patients received ERAS care and open surgery, while 80 patients received TC in laparoscopic surgery. Finally, 237 patients received ERAS care and laparoscopic surgery.

### Characteristics of included studies

Table 1 shows the general characteristics of included studies. Baseline characteristics within studies did not differ. The studies included patients with adenocarcinoma or adenoma of the colon or rectum [22, 25, 26] or patients undergoing elective colorectal surgery [21, 28, 30, 33] with indications such as malignant or benign disease, Crohn’s disease, ulcerative colitis and diverticular disease.

**Table 1:** Age is presented as median, unless mentioned different

Study	Design		*N* included	Age (years)	Sex (% male)	ASA I or II (%)
Vlug [42]	RCT, factorial 2 × 2 design laparoscopic ± ERAS open ± ERAS	ERAS (open/lap)	93/100	66/66 (mean)	58/53	81/82
		TC (open/lap)	98/109	66/68 (mean)	60/62	77/80
Basse [21]	RCT, ERAS open/laparoscopic	Open	30	75 (57–90)	53	63
		Lap.	30	75.5 (58–85)	53	83
King [22]	RCT, ERAS open/laparoscopic	Open	19	70 (mean)	42	84
		Lap.	41	72 (mean)	56	80
Al Chalabi [26]	Non randomised CCT, laparoscopic ERAS/TC	ERAS	37	53.9	51	57
		TC	36	61	67	64
Lloyd [28]	Non randomised CCT laparoscopic with ERAS/TC	ERAS	55	ns	ns	ns
		TC	15	ns	ns	ns
MacKay [29]	CCT, ERAS open/laparoscopic	Open	58	73 (67–82)	43	74
		Lap.	22	72 (64–79)	55	77
Junghans [27]	CCT, ERAS open/laparoscopic	Open	47	67 (31–84)	57	53
		Lap.	100	65 (32–76)	48	67
Raue [30]	CCT, laparoscopic ERAS/TC	ERAS	23	63 (32–76)	35	52
		TC	29	65 (38–86)	66	72

*CCT* controlled clinical trial, *RCT* randomised controlled trial, *TC* traditional care, *lap.* laparoscopic, *ns* not specified

### Number of ERAS items used

The number of ERAS items used in ERAS protocols as described by authors of included studies was analysed. This is depicted in Table 1. The included RCTs used a high number of ERAS items while some of the CCTs only described eight. Since this is more than the required seven ERAS items, these studies were included but could represent some bias in the studies. However, not all items were explicitly described, so the actual number of ERAS items could be higher.

### Methodological quality of studies

**Fig. 2:**
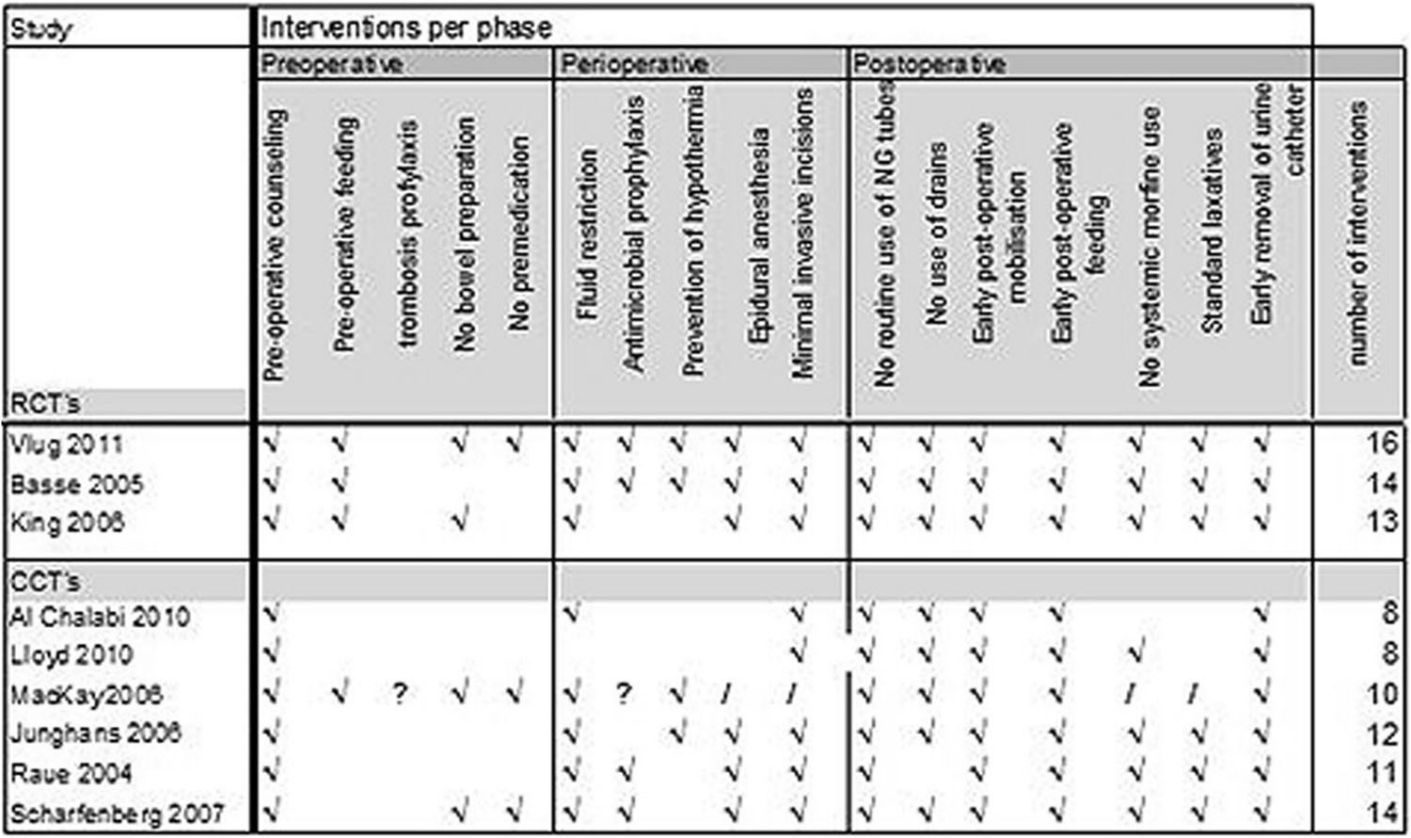
ERAS items used in included studies

**Fig. 3:**
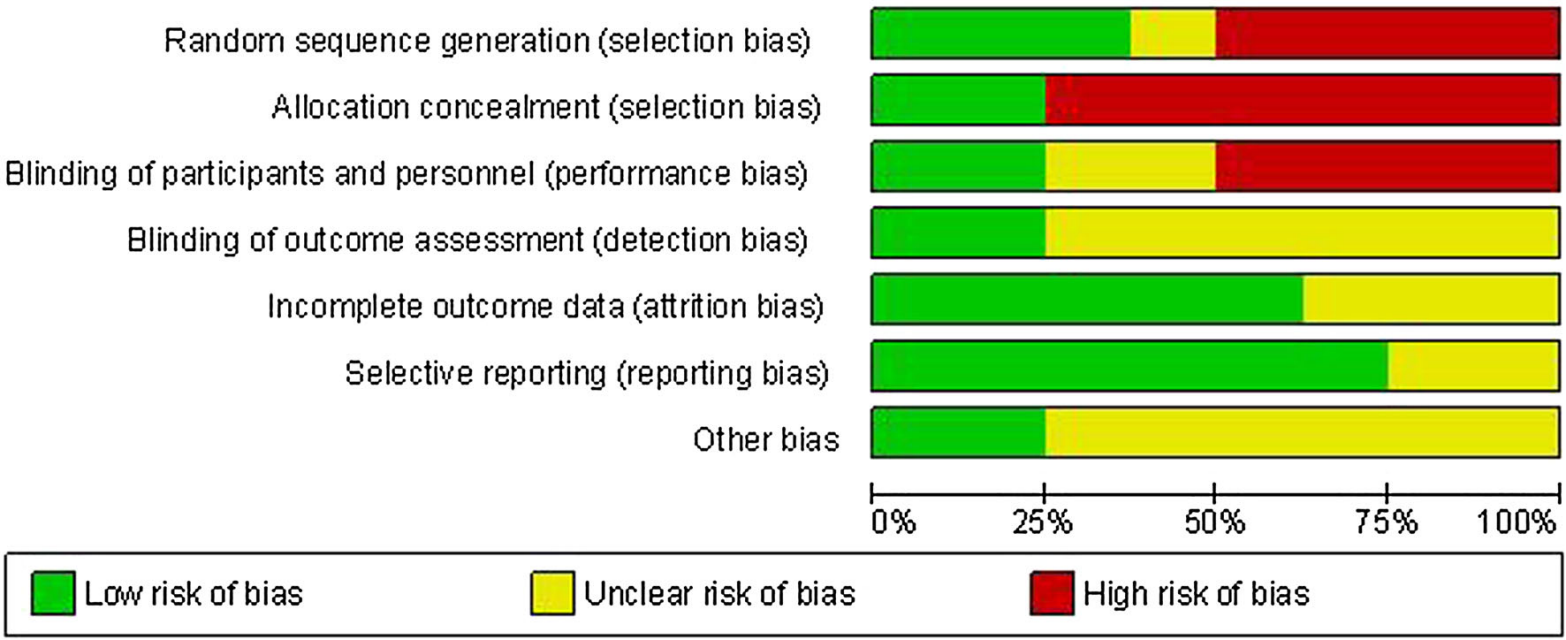
Risk of bias table according to grade

**Fig. 4:**
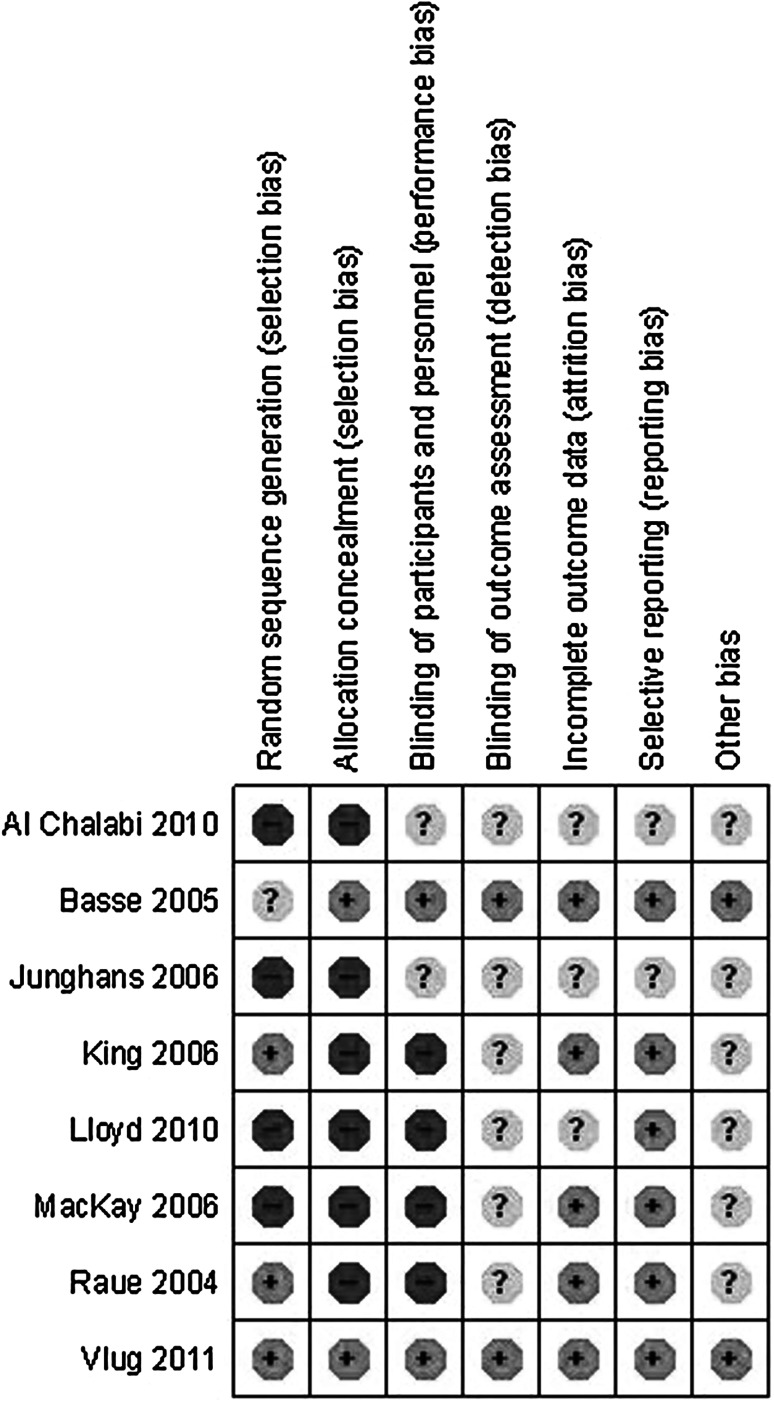
Bias items per study according to grade

The quality of included studies was analysed using GRADE methodology as described earlier. This is graphically depicted in Figs. 2 and 3. The most recent RCT by Vlug et al. was well devised and executed. This resulted in low risk of bias. The older RCTs, however, as well as the CCTs displayed far more risk of bias. In particular, the randomisation technique was not well described in the RCTs and of course absent in the CCTs. But allocation concealment and blinding were not used well. This is partially due to the studied intervention. ERAS is largely dependent on knowledge of both patients and staff concerning the intervention. Therefore, blinding is not possible. However, the use of open or laparoscopic techniques can be blinded. Other items were not well described resulting in unclear risk of bias. Overall, the methodological quality of included studies could be considered moderate to poor (Fig. 4).

### Primary outcome parameters

#### Morbidity

##### ERAS versus conventional within laparoscopy

When looking at complications in all, no significant differences between groups were noted. A subdivision in major complications also showed no difference between groups, nor did minor complications (Table 2).

**Table 2:** Summation of forest plots

Laparoscopic ± ERAS groups	ERAS	Conv	ERAS	Conv	Odds ratio	95 % CI	*I* ^2^ (%)	Effect (*Z*)	*p*
Comparison	Total *n*		Events						
All complications	215	189	58	54	0.97	[0.62; 1.53]	0	0.11	0.91
Major complications	215	189	24	24	0.95	[0.51; 1.75]	0	0.17	0.86
Minor complications	215	189	49	53	0.84	[0.52; 1.36]	0	0.72	0.47
Mortality	215	189	3	3	1.05	[0.21; 5.30]	0	0.06	0.95
Readmissions	160	174	11	11	1.08	[0.46; 2.57]	0	0.18	0.86
Postoperation hospital stay	215	189	NA	NA	–2.34^a^	[–3.77; –0.91]^a^	83	3.20	0.001

ERAS; laparoscopic versus open	Laparoscopic	Open	Laparoscopic	Open	Odds ratio	95 % CI	*I* ^2^ (%)	Effect (*Z*)	*p*
Comparison	Total *n*		Events						
All complications	252	228	93	99	0.62	[0.38; 1.01]	30	1.94	0.05
Major complications	271	189	43	57	0.42	[0.26; 0.66]	15	3.73	0.0002
Minor complications	130	123	45	48	1.63	[0.16; 16.31]	86	0.42	0.68
Mortality	193	200	4	9	0.51	[0.16; 1.70]	0	1.09	0.27
Readmissions	193	200	14	22	0.54	[0.27; 1.10]	0	1.71	0.09
Postoperation hospital stay	293	247	NA	NA	–0.40^a^	[–1.70; 0.91]	73	0.59	0.55

^a^Mean difference in days, *conv* conventional

##### Laparoscopy versus open within ERAS

**Fig. 5:**
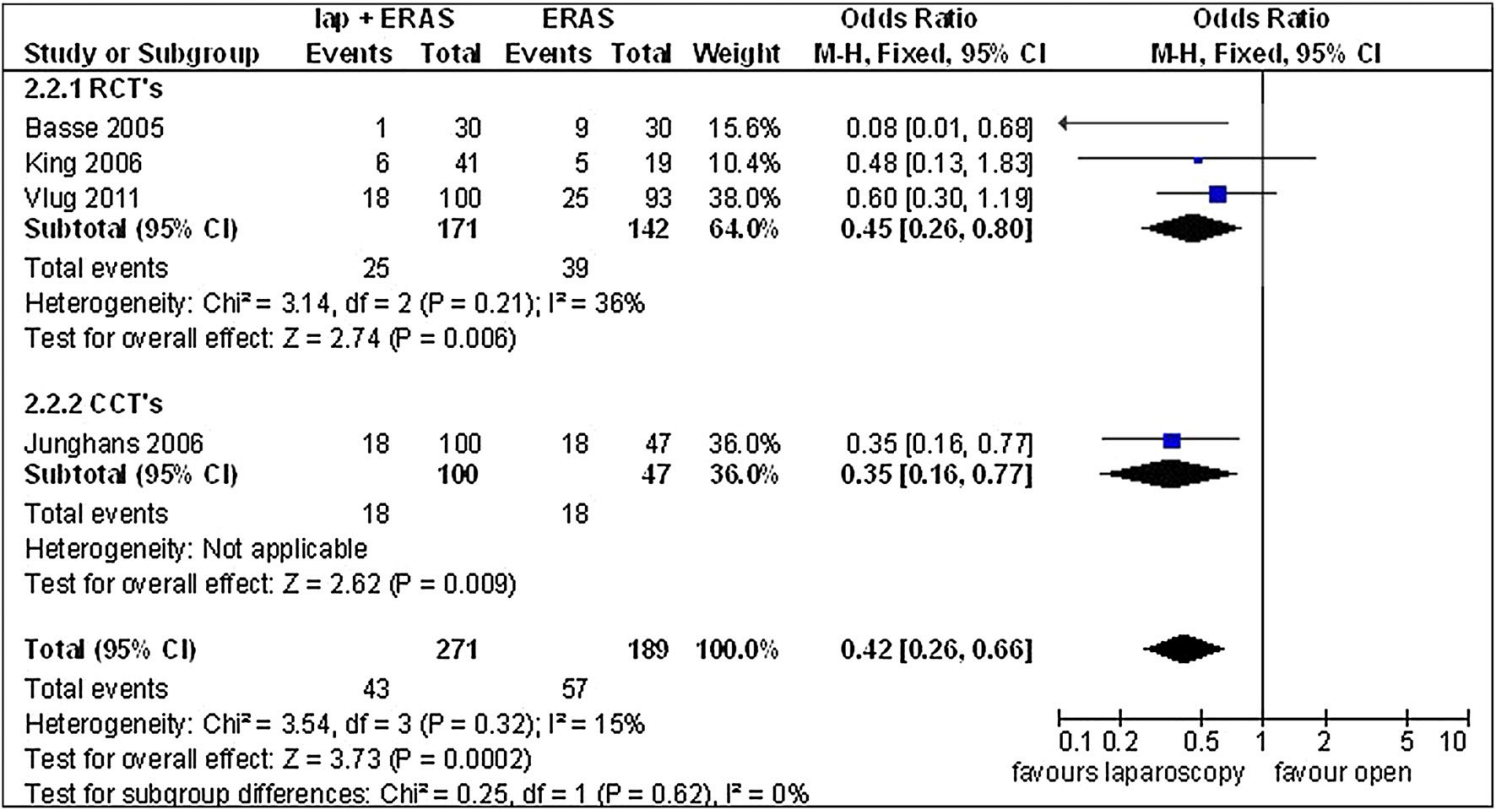
Forest plot of comparison: laparoscopic versus open surgery within ERAS, outcome: complications, major

Overall, complications did not differ between groups (Table 2). One study, however, did not report on overall morbidity, but only reported major morbidity [22]. When looking at major morbidity, a significant difference did exist, favouring laparoscopy (Table 2; Fig. 5) (OR 0.42 [0.26, 0.66], *Z* = 3.73, *p* = 0.006). Minor morbidity was not adequately reported in one RCT [22] and all of the CCTs [27, 29]. No difference between groups was noted for minor morbidity.

#### Mortality

##### ERAS versus conventional within laparoscopy

Studies reported low mortality, comparable to literature. No significant differences between groups were found (Table 2).

##### Laparoscopy versus open within ERAS

Mortality was not reported in one CCT [27]. Other studies reported relatively low mortality, comparable to literature. There was no difference between groups (Table 2).

#### Readmissions

##### ERAS versus conventional within laparoscopy

For the ERAS versus conventional groups within laparoscopic patients, 11 patients were readmitted in the ERAS population and 11 patients in the conventional group (OR 1.08 [0.46, 2.57]) (Table 2).

##### Laparoscopy versus open within ERAS

Readmissions did not differ significantly for both groups. For the laparoscopic versus open groups within ERAS protocols, 14 patients were readmitted in the laparoscopic groups versus 22 in the open groups (OR 0.54 [0.27, 1.10]) (Table 2).

Two studies did not adequately report readmissions [27, 28]. Both were CCTs. All readmissions occurred within 30 days.

#### Length of hospital stay

Length of hospital stay can be divided into postoperative hospital stay (PHS) and total hospital stay (THS), which is made up by PHS + days spent in hospital after readmission. Most studies only reported on PHS and not described the THS.

##### ERAS versus conventional within laparoscopy

**Fig. 6:**
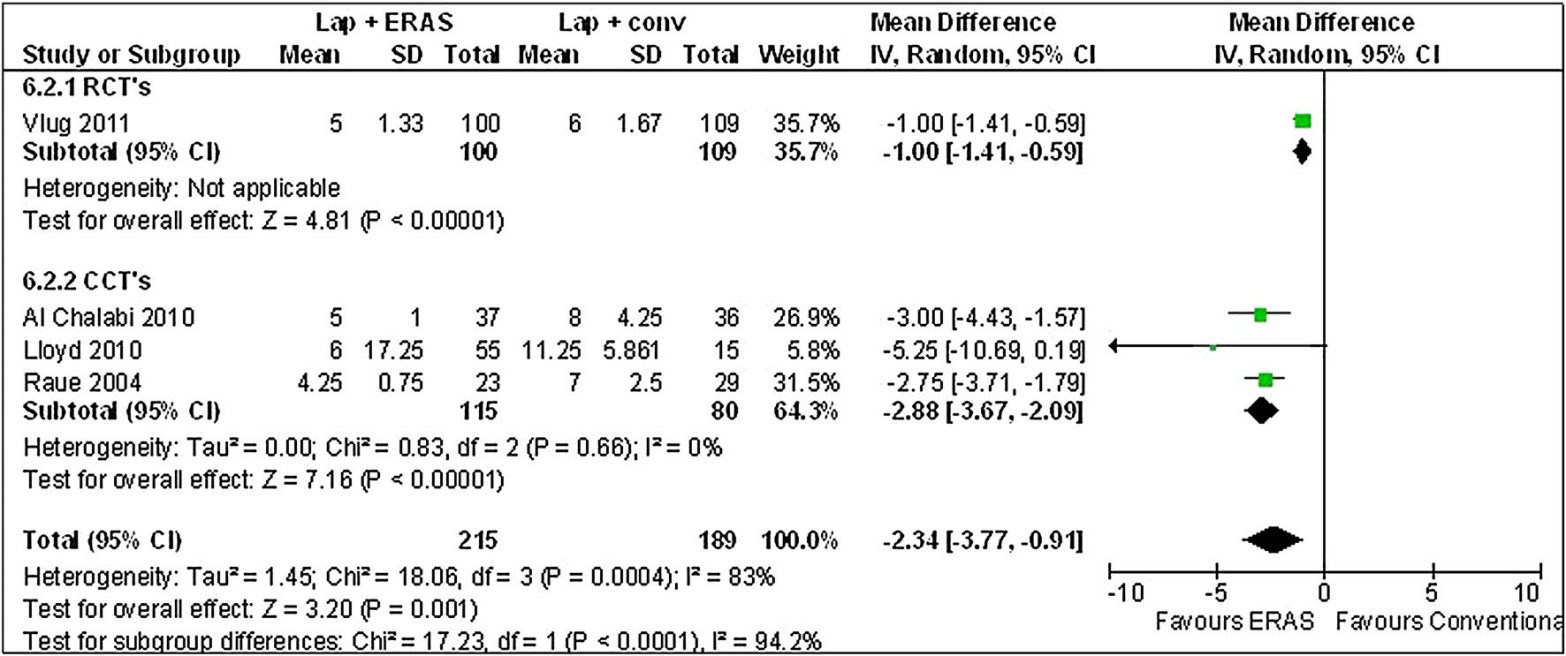
Forest plot of comparison: laparoscopy ± ERAS, outcome: postoperative hospital stay

PHS favours ERAS when using laparoscopic surgery (Table 2; Fig. 6) (MD –2.34 [−3.77, −0.91], *Z* = 3.20, *p* = 0.001). THS was only reported by Vlug et al. [25], so no meta-analysis could be performed. However, Vlug also favoured ERAS (MD −1.00 [−1.20, −0.80], *p* < 0.00001).

##### Laparoscopy versus open within ERAS

When looking at PHS between open and laparoscopic surgery when using ERAS, no differences between groups were found (MD −0.40 [−1.70, 0.91], *Z* = 0.59, *p* = 0.55). THS was not reported well. Basse et al. did report a mean difference without SD, which showed no significant difference between groups. Vlug et al. did report THS which favoured the use of laparoscopy (MD −2.00 [−2.24, 1.76], *p* < 0.00001).

### Secondary outcome parameters

#### Quality of life

Three of the included studies analysed quality of life. Vlug et al. [25] analysed quality of life 2 and 4 weeks postoperatively. No differences existed between groups. King et al. [22, 23] also found no differences in postoperative quality of life between open and laparoscopic ERAS groups. However, only 58 % of the open patients felt fully recovered 12 months after surgery versus 88 % of laparoscopic patients. Mackay et al. [29] also found no differences in quality of life between open and laparoscopic ERAS patients.

#### Gastrointestinal function

Several studies reported on gastrointestinal function. The way this was reported did, however, differ significantly between studies. Van Bree et al. [24] reported on gastrointestinal function in the LAFA trial. Gastric emptying and GI transit were investigated by scintigraphy. Gastric emptying did not differ, but colonic transit was significantly faster in the laparoscopic ERAS group. Clinically, this did not result in earlier tolerance for food or passage of stool. Basse [21] and Raue [30] also reported on gastrointestinal function and found no differences.

#### Pain and pain medication

Basse et al. [21] reported that laparoscopic patients actually had a slightly higher pain score on day 0 and 1. This difference disappeared on day 2. Other studies reported no differences between groups [29, 30]. In the study by King, significantly more patients needed additional opioids in the open group [34]. Not all studies routinely used thoracic epidural analgesia in their ERAS protocols.

#### Cost

In hospital, cost did not differ between groups in the LAFA study, despite the fact that hospital stay was reduced in both the open ERAS and laparoscopic ERAS groups [25]. King et al. [22] reported that laparoscopic surgery was more expensive than open surgery, but total cost did not significantly differ.

### Other outcome parameters

No differences were reported for several outcome parameters, like motor function, pulmonary function and mental function.

#### Protocol compliance

Vlug et al. [25] reported on protocol compliance. They found that out of the analysed 15 elements, the laparoscopic ERAS group had a compliance of 11.2 ± 2.2 items. The open ERAS group complied with 11.1 ± 2.2 items, while the laparoscopic and open groups with standard care used 6.0 ± 1.5 and 5.8 ± 1.4 items, respectively. None of the other studies included reported on protocol compliance.

## Discussion

The aim of this review was to investigate whether laparoscopic surgery and ERAS have additional, i.e. synergistic value within colorectal surgery. The systematic search resulted in three RCTs with 520 patients and five CCTs, including 422 patients. In all, 408 patients received laparoscopic ERAS care, 189 patients received laparoscopic conventional care, and 247 received open ERAS care.

When comparing ERAS and conventional care within laparoscopic surgery, no differences in complications were found. In contrast, a Cochrane review showed reduced complication rates when ERAS was applied in open surgery [7]. Therefore, laparoscopy might offer additional advantages that cancel out ERAS advantages in complication rates. To date, large RCTs for laparoscopic versus open colon surgery showed no differences in complication rates [35, 36]. However, in these studies, postoperative care was not standardised and not a part of the studied parameters. Additionally, a Cochrane review, including 25 RCTs with 3526 participants, did show a significant reduction in postoperative complications favouring laparoscopic surgery [3].

Other primary outcome parameters also showed no differences. Readmission numbers did not differ between groups, but did differ between RCTs, ranging between 20 versus 27 % for laparoscopic and 6 and 8 % for open surgery groups, respectively. These readmission numbers are higher than in other trials concerning ERAS care in open surgery [7]. Postoperative hospital stay when comparing ERAS and conventional care in laparoscopic surgery was shorter in the ERAS group (MD −2.34 [−3.77, −0.91], *Z* = 3.20, *p* = 0.001). Therefore, since complications did not differ, ERAS does improve length of stay in laparoscopic surgery.

When comparing laparoscopic and open surgery within ERAS postoperative care, a significant reduction in major complications favouring laparoscopy was found (OR 0.42 [0.26, 0.66], *Z* = 3.73, *p* = 0.006). This is in line with the earlier statement that laparoscopy offers additional advantages in complication reduction. Overall morbidity, readmissions and mortality showed no differences. When comparing laparoscopic and open surgery with ERAS postoperative care, no differences for postoperative hospital stay between groups were found (MD −0.45 [−1.53, 0.63], *Z* = 0.81, *p* = 0.42).

For hospital stay, however, a lot of heterogeneity was present, with mean length of stay ranging from 2.3 to 7 days between studies and even differed within groups. Therefore, these results might be biased by the primary goal of included studies, as one study had a planned discharge of 2 days postoperative [21]. No studies reported on the factor “fulfilling discharge criteria”, which could be a more sensitive parameter for speed of recovery, because it is not influenced by non-medical factors like logistics.

A well-known problem with implementing ERAS programmes and maintaining them is protocol compliance [32, 37, 38]. Implementing some aspects of ERAS into regular care and failure to enforce items within the ERAS programme could influence results. In this review, only one study reported on protocol compliance [25]. ERAS compliance was around 11 items, while conventional care also used around six items. This could have impacted differences in outcome. Other studies did not report on protocol compliance, but the number of ERAS items used in the respective protocols also differed greatly, while all studies seemed to also incorporate some ERAS items in the conventional group. This could be a large source of bias, especially in comparison between ERAS and conventional care.

Secondary outcome parameters showed no differences between groups. Cost, an often-mentioned factor against implementing laparoscopic surgery within standard (=ERAS) colorectal surgical care programmes [39, 40], did not differ between groups either.

### Bias

Methodological quality of studies was moderate to poor overall, with exception of the LAFA trial [25]; especially blinding of outcome assessors and clinicians was lacking. This could have resulted in observer bias, especially for outcome parameters like hospital stay. Time lag bias could have been introduced by the fact that trials significantly diverge in the time frame included trials were undertaken. Earliest trials are published in 2004, with inclusion in the early 2000s. The latest trial was published in 2011 and included patients after 2009.

Not all studies included used high number of ERAS items in their protocols, and some items (especially thoracic epidural analgesia) were used in the conventional protocol. Although all included studies satisfied the condition of using at least seven items in the ERAS protocol, this ranged between eight and almost all items. This could have introduced bias concerning the exact effect of ERAS as opposed to conventional care.

The combination of these methodological limitations and bias could have influenced meta-analysis outcome and also the conclusions for clinical implications.

### Other studies

The systematic search also identified several either retrospective or non-comparative studies concerning laparoscopic surgery with or without ERAS care. Feroci et al. [11] published a retrospective analysis of a prospective database including 209 patients receiving laparoscopic surgery with ERAS care and 141 patients with open ERAS surgery. Length of stay was reduced in the laparoscopic group, as were non-surgical complications, time to diet tolerance, bowel movement and length of analgesia administration. This study therefore favours laparoscopic surgery.

Tsikitis et al. included 197 patients in a retrospective study comparing laparoscopic surgery with (*n* = 82) and without (*n* = 115) ERAS aftercare. Length of stay and complications were significantly reduced in favours of ERAS care. Other studies also favoured laparoscopy with ERAS care [10, 41]. However, since all of these studies comprised of retrospective studies a large risk of bias impedes interpretation of these results.

The search also resulted in two ongoing studies. The first is the TAPAS trial, a prospective cohort trial (*n* = 225 patients) comparing open surgery and conventional care, open surgery and ERAS and laparoscopic surgery and ERAS care for colon carcinoma [42]. Results are expected shortly. The second trial is the EnROL trial, a phase III, multicentre, randomised trial of laparoscopic versus open resection of colon and rectal cancer with blinding of patients and outcome observers to the treatment allocation [43]. Both are well designed with adequate power that could influence results of a future update of this meta-analysis.

## Conclusions

This meta-analysis investigated the possible additional, i.e. synergistic effects of laparoscopy and ERAS in colorectal surgery care. Results show a significant reduction in major morbidity in favour of laparoscopy compared to open surgery within ERAS care while there was no difference in complications between conventional care and ERAS in the laparoscopic group. We therefore conclude that laparoscopy has a (major) additional effect within ERAS care, especially because postoperative hospital stay was significantly reduced in the laparoscopic group with ERAS care. However, since the limitations of this meta-analysis included small sample sizes, high risk of bias due to lacking methodological quality and lack of protocol implementation control, conclusions should be regarded with some reservations. Better designed large trials are needed, and two ongoing trials were identified.
